# Self-Assembled Nanocomposite DOX/TPOR_4_@CB[7]_4_ for Enhanced Synergistic Photodynamic Therapy and Chemotherapy in Neuroblastoma

**DOI:** 10.3390/pharmaceutics16060822

**Published:** 2024-06-18

**Authors:** Zhouxia Lu, Xu Chen, Conghui Wang, Xuelian Luo, Xiaohan Wu, Xing Zhao, Song Xiao

**Affiliations:** 1 Department of Chemistry, School of Basic Medicine, Guizhou Medical University, Guiyang 550025, China; lzx5882@sina.com (Z.L.); pandaxxu@outlook.com (X.C.); wch1515689971@sina.com (C.W.); 15121604241lxl@sina.com (X.L.); qq2824836751@sina.com (X.W.); 2 Department of Histology and Embryology, School of Basic Medicine, Guizhou Medical University, Guiyang 550025, China; 3 Tumor Immunotherapy Technology Engineering Research Center, Guizhou Medical University, Guiyang 5500025, China; xingzhao@gmc.edu.cn

**Keywords:** porphyrin, photodynamic therapy, singlet oxygen, apoptosis

## Abstract

DOX/TPOR_4_@CB[7]_4_ was synthesized via self-assembly, and its physicochemical properties and ability to generate reactive oxygen species (ROS) were evaluated. The impact of photodynamic therapy on SH-SY5Y cells was assessed using the MTT assay, while flow cytometry analysis was employed to detect cell apoptosis. Confocal laser scanning microscopy was utilized to observe the intracellular distribution of DOX/TPOR_4_@CB[7]_4_ in SH-SY5Y cells. Additionally, fluorescence imaging of DOX/TPOR_4_@CB[7]_4_ in nude mice bearing SH-SY5Y tumors and examination of the combined effects of photodynamic and chemical therapies were conducted. The incorporation of CB[7] significantly enhanced the optical properties of DOX/TPOR_4_@CB[7]_4_, resulting in increased ROS production and pronounced toxicity towards SH-SY5Y cells. Moreover, both the apoptotic and mortality rates exhibited significant elevation. In vivo experiments demonstrated that tumor growth inhibition was most prominent in the DOX/TPOR_4_@CB[7]_4_ group. π–π interactions facilitated the binding between DOX and photosensitizer TPOR, with TPOR’s naphthalene hydrophilic groups encapsulated within CB[7]’s cavity through host–guest interactions with CB[7]. Therefore, CB[7] can serve as a nanocarrier to enhance the combined application of chemical therapy and photodynamic therapy, thereby significantly improving treatment efficacy against neuroblastoma tumors.

## 1. Introduction

Neuroblastoma (NB) is an embryonic tumor of the sympathetic system, originating from 8% to 10% of tumors in children. It represents the most prevalent malignant solid neoplasm in infants and young children, ranking as the second most common extracranial malignancy among this age group [1,2,3]. Neuroblastoma displays remarkable phenotypic heterogeneity [4]. High-risk patients have a dismal prognosis, with a survival rate below 50%, even after undergoing chemotherapy, radiotherapy, and surgery [5,6]. Consequently, there is an urgent need to develop innovative and reliable therapeutic strategies for enhancing neuroblastoma treatment.

Due to the multidrug resistance exhibited by tumor cells and the inherent heterogeneity of tumors, monotherapy alone is insufficient for the complete eradication of tumors. A complementary strategy involving the combination of multiple therapies with distinct mechanisms is imperative [7,8,9]. Photodynamic therapy (PDT) is a clinically approved noninvasive therapeutic modality of significant importance [10]. PDT is regarded as an optimal noninvasive modality that offers precise tumor treatment with simplicity, flexibility, and temporal control [11,12,13]. The utilization of photosensitizers (PS) enables the generation of cytotoxic reactive oxygen species (ROS), particularly highly reactive singlet oxygen (^1^O_2_), which effectively interacts with adjacent biological macromolecules, thereby inducing cellular apoptosis [14,15]. However, its photodynamic effect is diminished due to its tendency to undergo self-aggregation [16,17,18].

Chemotherapy, the most prevalent approach for cancer treatment, exhibits less invasiveness compared to surgery or radiation, exerts relatively minimal overall adverse effects on patients, and demonstrates remarkable efficacy in eradicating tumor cells and preventing metastasis [19,20,21]. However, this statement highlights the substantial toxicity and adverse effects on the human body associated with chemotherapy, as well as its propensity to develop drug resistance. In contrast, combination therapy has been shown to elicit synergistic effects when compared to monotherapy [22,23,24]. Despite its immense potential in tumor treatment, combination therapy has not been fully realized due to factors such as toxic side effects of drugs and cell uptake efficiency.

The advancement of molecular self-assembly technology has attracted significant attention from researchers, particularly in the field of nanodrug carriers [25,26]. To enhance the solubility, stability, and bioavailability of drugs in biological systems, numerous scientific research groups are employing principles of supramolecular chemistry to design complexes based on various supramolecular interactions including ion–dipole, hydrogen bonding, dipole–dipole, and hydrophobic interactions between host and guest molecules [27,28,29]. The cucurbit[n]urils represent a novel and biocompatible supramolecular drug carrier [30]. The macrocyclic cage-like compounds are interconnected by methylene bridges and consist of 2n methylene units bridging with glycoluril moieties. Due to the distinctive structure featuring rigid hydrophobic cavities of varying sizes and hydrophilic ports in cucurbit[n]urils, highly selective and stable host–guest inclusion complexes can be formed with a diverse range of guest molecules [31,32,33]. The moderate water solubility of CB[7] (2~3 × 10^−2^ mol/L) facilitates its versatile application in drug delivery. Moreover, the low toxicity of CB[7] enables its wide applicability, wide applicability due to the formation of drug-CB[7] complexes, which enhance chemical and physical stability, improve solubility, increase bioavailability, and allow for controlled release of drugs [34,35,36]. Numerous studies have demonstrated that the utilization of cucurbit[n]uril as a carrier can effectively enhance the efficacy of photodynamic therapy while simultaneously mitigating the toxicity and adverse effects associated with the photosensitizer itself [37,38,39]. Our team has proposed a new approach using macrocycle-anchored polymer-triggered vesicles, which were prepared from one-pot processed CB[7]-anchored polymers to enhance photosensitization [40]. The water-soluble porphyrin photosensitizer, 5,10,15,20-tetra-(4-pyridinyl, N-β-bromomethylnaphthalene) porphyrin (TPOR), was successfully synthesized with excellent solubility in aqueous solutions by our team [41].

The drug DOX not only demonstrates broad-spectrum antitumor activity but also serves as a radiosensitizer for the treatment of neuroblastoma. A water-soluble porphyrin photosensitizer, 5,10,15,20-tetra (4-pyridyl, N-β-brommethyl naphthalene) porphyrin (TPOR) has high solubility in aqueous environments. In this study, our objective was to enhance the efficacy of tumor photodynamic therapy by designing nanodrugs DOX/TPOR_4_ for combined chemotherapy treatment. We employed a simple and efficient method to encapsulate them into the carrier CB[7], resulting in the preparation of the DOX/TPOR_4_@CB[7]_4_ double-loading compound. Remarkably, the self-assembled DOX/TPOR_4_@CB[7]_4_ drug exhibited outstanding optical performance, enhanced ROS production, reduced side effects, and significantly improved neuroblastoma efficacy. Importantly, our nanoplatform integrates photodynamic therapy and chemotherapy into a unified platform, thereby maximizing treatment effectiveness.

## 2. Materials and Methods

### 2.1. Chemical Synthesis and Characterization

#### 2.1.1. Preparation of DOX/TPOR_4_@CB[7]_4_

The TPOR, DOX, and CB[7] were individually dissolved in DMSO and subjected to 4 h of ultrasonication under light-avoidance conditions. The TPOR and DOX were individually stirred for 12 h under conditions of light avoidance. The DOX solution was gradually introduced into the TPOR solution and agitated for 24 h under light-avoidance conditions to obtain the DOX/TPOR_4_ solution, then added into the solution of CB[7] in definite proportion with 1:4. Characterization of ultraviolet–visible spectra and fluorescence spectra were detected by ultraviolet spectrophotometer (UV-2600, Shimadzu, Kyoto, Japan) and fluorescence spectroscopy (Cary eclipse, VRIAN, Santa Clara, CA, USA).

#### 2.1.2. Morphological Characteristics

DOX/TPOR_4_@CB[7]_4_ was synthesized in the same way, with methanol as the solvent. It was then dried by a freeze-drying machine to form a solid powder for scanning electron microscope (SEM NANO450, FEI) and transmission electron microscope (TEM JEM2100F, Tokyo, Japan) observation.

#### 2.1.3. Hydrate Particle Size and Zeta Potential

The TPOR, DOX/TPOR_4_, DOX/TPOR_4_@CB[7]_4_ was diluted in pure water to 10 μM concentration working solution. A Particle Tracking Analyzer (Zeta View, PMX, Munich, Germany) detected water, particle size, and Zeta potential.

### 2.2. Detection of Singlet Oxygen in Solution

The Singlet Oxygen Sensor Green (SOSG) reagent (Invitrogen, Waltham, MA, USA) working solution was mixed with TPOR, DOX, DOX/TPOR_4_, and DOX/TPOR_4_@CB[7]_4_ in water, and irradiated with 525 nm 95.5 mW/cm^2^ laser for different time. After illumination, it was determined by fluorescence spectroscopy (Cary Eclipse, VRIAN, USA).

### 2.3. Cells and Animals

Neuroblastoma cells SH-SY5Y was obtained from the American Type Culture Collection (ATCC) and cultured in DMEM/F12 (Gibco, Grand Island, NY, USA) supplemented with 10% fetal calf serum (Every Green, Shenzhen, China), and 1% penicillin-streptomycin (Gibco, USA). Cells were maintained at 37 °C in a 5% CO_2_ incubator. Healthy female BALB/c-nude mice (4–6 weeks old) were purchased from SPF (Beijing, China) Biotechnology Co, Ltd. Tumor-bearing mice were constructed by subcutaneously injecting a suspension of 1 × 10^7^ SH-SY5Y cells. All mice procedures were approved by and raised in the SPF laboratory animal room.

### 2.4. Intracellular ROS Detection

To detect the intracellular ROS, we used the 2,7,-Dichlorodi-hydrofluorescein diacetate (DCFH-DA) as a probe. Collected cells were inoculated into confocal culture dishes (φ15 mm glass dish, 8 × 10^4^ cells/dish) and cultured for attachment at 37 °C in 5% CO_2_ incubator. Then, incubated with 2.5 μM TPOR, DOX/TPOR_4_, DOX/TPOR_4_@CB[7]_4_ and 0.625 μM of DOX for 24 h. Cells were washed three times, and DCFH-DA (10 μM) was added and incubated for 30 min. 525 nm laser was used to illuminate with 95.5 mW/cm^2^ for different periods. The fluorescence images of the cells were observed by the Confocal laser scanning microscope (CLSM, Nikon, Kyoto, Japan). The mean density was analyzed by Image-Pro Plus 6.0.

### 2.5. Drug Cellular Localization Test

The cells were seeded in confocal culture dishes (φ15 mm glass dish) and cultured for attachment. The cells were incubated with 2.5 μM TPOR, DOX/TPOR_4_, DOX/TPOR_4_@CB[7]_4_, and 0.625 μM DOX for 24 h. After being washed with PBS three times, DAPI (Solarbio, Beijing, China) was added. After incubation for 30 min, it was washed with PBS for five times. After incubation with MitoTracker Green for 45 min, it was washed with PBS for three times and imaged with CLSM.

### 2.6. Cell Viability Assay

The cells were seeded in a 96-well plate (4 × 10^4^ cells/well) and cultured for attachment. Cellular viability was assessed using the MTT [(3-(4, 5-dime-thylthiazol-2-yl)-2, 5-diphenyltetrazolium bromide] colorimetric assay.

#### 2.6.1. Toxicity without Irradiation

DOX, TPOR, DOX/TPOR_4_, DOX/TPOR_4_@CB[7]_4_ was added into different concentrations (1, 2.5, 5, 10 μM), respectively, for 24 h, and washed with PBS three times. Then, the fresh DMEM medium was replaced and incubated for 24 h. An MTT assay was used to determine the cell viability rate with an ultramicroporous plate spectrophotometer (Epoch2, Biotek, Santa Clara, CA, USA).

#### 2.6.2. In Vitro Synergistic Therapeutic Effects of PDT and Chemotherapy

The cells were incubated with 0.625 μM DOX and 2.5 μM CB[7], TPOR, DOX/TPOR_4_, DOX/TPOR_4_@CB[7]_4_ for 24 h. Then, the cells were replaced with fresh DMEM medium and irradiated with 525 nm laser for 0.5 min, 1 min, 2 min and 3 min at 95.5 mW/cm^2^ intensity in the dark room. The survival rate of the cells was measured by MTT assay.

### 2.7. Flow Cytometry

Cell death was assessed post-PDT using the Apoptosis Assay Kit (KeyGEN, Nanjing, China). Treated and control samples were analyzed using FlowJo VX on the BD FACS Celesta (BD, San Jose, CA, USA).

### 2.8. In Vivo Targeting Ability Evaluation

The in vivo tumor-targeting ability of DOX/TPOR_4_@CB[7]_4_ was evaluated by fluorescent imaging on an IVIS Spectrum system (PerkinElmer, Waltham, MA, USA). Briefly, 2.5 μM DOX and 10 μM TPOR DOX/TPOR_4_ and DOX/TPOR_4_@CB[7]_4_ were intravenously injected into the tumor bearing mice and the fluorescent images of mice at intervals of 0 h, 12 h, 24 h, 36 h, and 48 h were observed and recorded.

### 2.9. In Vivo Anti-Tumor Evaluation

The tumor-bearing mice were randomly divided into four groups (n = 6 mice/group) with treatments of PBS + Light, TPOR + Light, DOX/TPOR_4_ + Light, and DOX/TPOR_4_@CB[7]_4_ + Light. These mice were injected for drug administration and subsequently exposed to darkness in a controlled environment, then received photodynamic therapy after 24 h (6 min, 150 mW/cm^2^). Every 3 days, drugs were injected to different treatments and received light irradiation after 24 h of intravenous injection according to the in vivo imaging results. Changes in tumor volume and mice body weight were recorded as well. After 18 days of treatment, the mice were euthanized and the harvested tumor tissues were fixed with 10% neutral formalin solution, followed by H&E staining and TUNEL staining analysis.

### 2.10. Statistical Analysis

All experiments were performed at least three times. Analysis was performed using SPSS Statistics 25.0. The one-way ANOVA test was used to compare the differences between multiple groups with significance accepted at *p* < 0.05.

## 3. Results

### 3.1. Characterization of DOX/TPOR_4_@CB[7]_4_

The self-assembly of nanodrugs in this study relied on the electrostatic interaction between drugs containing charged groups. TPOR and CB[7] can be combined through self-assembly, and their association constant was determined to be 3.15 (±0.23) × 10^5^ mol·L^−1^, indicating a strong interaction between TPOR and CB[7], resulting in the formation of a stable host–guest complex [41]. Nanodrugs DOX/TPOR_4_ were synthesized based on the electrostatic interactions between DOX with positively charged NH_2_ groups and the photosensitizer TPOR with negatively charged Br-groups. Additionally, we prepared DOX/TPOR_4_@CB[7]_4_ by combining DOX and TPOR through π–π conjugation and incorporating carrier CB[7] via self-assembly. TPOR possesses a conjugated aromatic π-system, which tends to undergo π–π stacking in aqueous media [42]. We prepared DOX/TPOR_4_@CB[7]_4_ by combining DOX and TPOR through π–π conjugation and incorporating carrier CB[7] via self-assembly.

**Figure 1:**
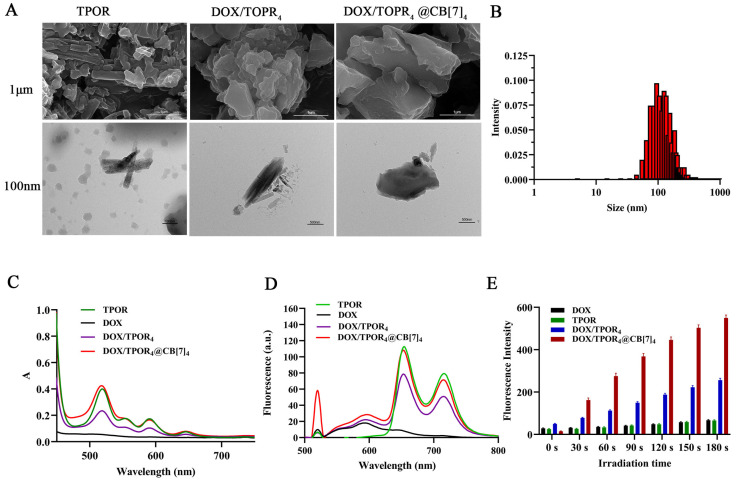
Characterization of DOX/TPOR_4_@CB[7]), (**A**) TEM and SEM images of TPOR, DOX/TPOR_4_ and DOX/TPOR_4_@CB[7]_4_; (**B**) the particle size distribution of DOX/TPOR_4_@CB[7]_4_; (**C**) images of the UV absorption spectrum and (**D**) fluorescence emission spectrum. (**E**) The fluorescence intensity changes of SOSG under 525 nm laser (95.5 mW/cm^2^) irradiation for different times (0 s, 30 s, 60 s, 90 s, 120 s, 150 s, 180 s) of DOX, TPOR, DOX/TPOR_4_, and DOX/ TPOR_4_@CB[7]_4_ (the drug concentration was 10 μM).

We then discussed the UV absorption spectra and fluorescence spectra of the different ratios of DOX, TPOR and CB[7]. We found that CB[7] had almost no absorption band in the UV–vis spectral range we were interested in. We observed a pronounced absorption band in the UV–vis spectral range of 500–700 nm for DOX/TPOR_4_-CB[7]_4_. Additionally, it exhibited intense fluorescence emission within the range of 600–800 nm (Figure S1) and the MTT assay demonstrated that photodynamic therapy exhibited the most significant efficacy (Figure S2), so DOX/TPOR_4_-CB[7]_4_ was used in subsequent experiments and named DOX/TPOR_4_@CB[7]_4_. The corresponding UV–vis absorption spectrum is shown in Figure 1C. DOX had no obvious visible absorption in the range of 500–700 nm. We used the concentration of TPOR (10 μmol/L) as the basis to measure UV–vis absorption intensity. The concentration of TPOR (10 μmol/L) was utilized as the reference for measuring UV–vis absorption intensity; the absorption intensity of DOX/TPOR_4_ was significantly lower than that of TPOR within the range of 600–800 nm. In addition, the absorption intensity of DOX/TPOR_4_@CB[7]_4_ was significantly increased and strongest of three compounds (A_TPOR_ = 0.4004, A_DOX/TPOR4_ = 0.2345, A_DOX/TPOR4@CB_[7] = 0.4248). The UV–vis absorption spectra data of DOX/TPOR_4_ and DOX/TPOR_4_@CB[7]_4_ revealed a slight blueshift (1–2 nm) in the absorption wavelength compared with TPOR. When excited at 520 nm, the fluorescence intensity of DOX/TPOR_4_ decreased significantly compared with TPOR, and there was fluorescence quenching. 

Moreover, the NH_2_ group of DOX has a positive charge, which could be combined with the negatively charged Br group of TPOR through electrostatic action to enhance the interaction relationship [43]. The fluorescence intensity of DOX/TPOR_4_@CB[7]_4_ was relatively enhanced compared with DOX/TPOR_4_. TPOR and CB[7] can be combined by self-assembled, and there was a strong interaction between TPOR and CB[7], which can form a stable host–guest complex [41]. The hydrophobic group of TPOR was fixed in the CB[7] cavity through host–guest interaction. The base nucleus of the porphyrin was large and remained outside the CB[7], which could still maintain the high concentration of porphyrin chromophore after combining with CB[7]. Combined with the previous results, it was preliminarily proved that the self-assembled DOX/TPOR_4_@CB[7]_4_ was formed [41]. It suggested that CB[7] could enhance fluorescence intensity. Therefore, high fluorescence emission was achieved in the DOX/TPOR_4_@CB[7]_4_ self-assembled nanosystem, which was conducive to observe in the cells and helpful for the visualization diagnosis and treatment of tumors.

After the addition of CB[7], the naphthalene hydrophobic groups of porphyrins in the compound were immobilized within the CB[7] cavity through host–guest interactions. The DOX/TPOR_4_@CB[7]_4_ assembly also exhibited a lamellar stacking morphology with a more densely packed arrangement and reduced surface irregularities, indicating successful formation between CB[7] and DOX/TPOR_4_ (Figure 1A). Size distribution analysis revealed that DOX/TPOR_4_@CB[7]_4_ was predominantly concentrated in the range of 100–200 nm, with an average particle size of 120.9 nm (Figure 1B). Furthermore, it can be observed that among these three entities, DOX/TPOR_4_@CB[7]_4_ displayed the highest Zeta potential (Figure S3B), with an average potential of 37.51 mV, demonstrating its relative stability. It was confirmed that DOX/TPOR_4_@CB[7]_4_ possessed superior physical and chemical properties. However, with CB[7] being water-soluble, it provides a hydrophobic cavity while having a hydrophilic exterior that offers numerous opportunities to enhance photosensitizer properties [44]. The further increase in particle size and zeta potential suggests successful loading of DOX/TPOR_4_ with CB[7], effectively preventing TPOR self-aggregation.

### 3.2. Revealing the Presence of Singlet Oxygen

In photodynamic effects, ^1^O_2_ plays a crucial role in inducing tumor cell death. The generation of ^1^O_2_ can be quantified by measuring its fluorescence intensity using a SOSG fluorescence probe. As illustrated in Figure 1E, the time-dependent SOSG fluorescence intensity of all four drugs demonstrates their ability to produce a certain amount of ^1^O_2_. Notably, when TPOR is combined with DOX, there is a significant enhancement in SOSG fluorescence prior to binding. Previous studies have reported that DOX sensitizes photosensitizer-mediated photodynamic therapy [45,46]. Consequently, DOX promotes the production of ^1^O_2_ when used in conjunction with TPOR. Among the tested samples, DOX/TPOR_4_@CB[7]_4_ exhibits the highest SOSG fluorescence intensity. After irradiation for 180s, the fluorescence intensity of DOX/TPOR_4_@CB[7]_4_ reaches as high as 600 units–approximately twice that of DOX/TPOR_4_ and six times that of TPOR alone. The addition of CB[7] significantly influences the production and output of ^1^O_2_. This discovery provides a robust foundation for potential applications in photodynamic therapy.

### 3.3. Reactive Oxygen Species(ROS) Quantification

**Figure 2:**
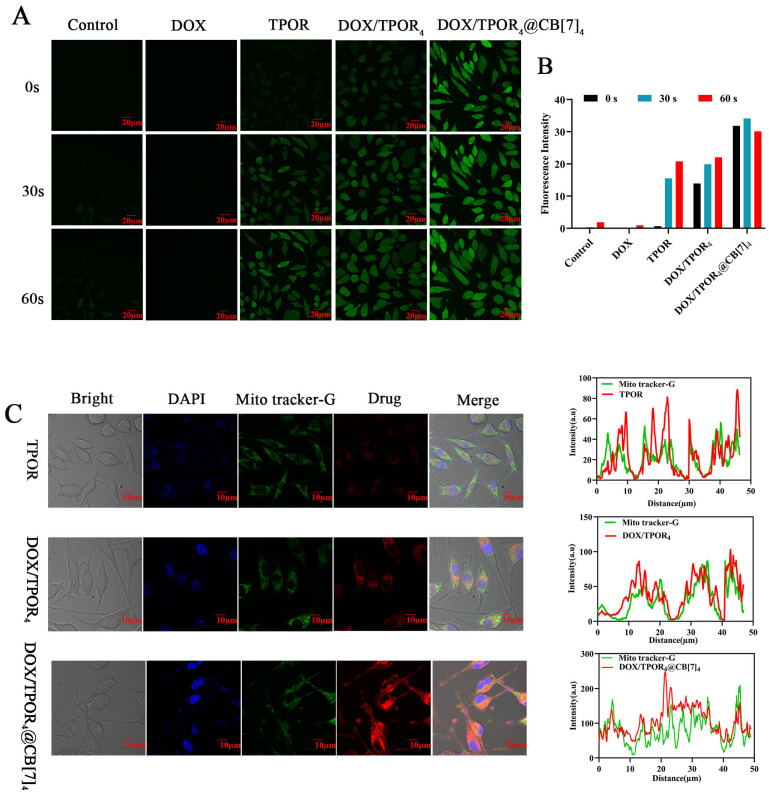
Uptake of drugs by SH-SY5Y cells and their ability to produce ROS. (**A**) ROS generation in SH-SY5Y cells of DOX, TPOR, DOX/TPOR_4_ and DOX/TPOR_4_@CB[7]_4_ groups after different illumination times (The drug concentration was 2.5 μM, Scale bar = 20 μm); (**B**) the statistical graph of ROS fluorescence changes; (**C**) intracellular distribution of DOX, TPOR, DOX/TPOR_4_ and DOX/TPOR_4_@CB[7]_4_ were conducted on SH-SY5Y cells after 24 h (The drug concentration was 5.0 μM, Scale bar = 10 μm).

Reactive oxygen species (ROS) play a key role in the photodynamic clearance of tumor cells [47]. DCFH-DA fluorescent probe was used to detect ROS, which oxidized to DCF and emitted green fluorescence [48]. As shown in Figure 2A, fluorescence was not observed in both the control group and the DOX group, indicating no ROS production. Under no light radiation, both DOX/TPOR_4_ and DOX/TPOR_4_@CB[7]_4_ exhibited obvious green fluorescence, and the fluorescence intensity of the latter was stronger. These experimental results in solution showed that DOX/TPOR_4_ produced a certain level of ROS (Figure 1E). Furthermore, TPOR can be activated to produce ROS even when exposed to a short period of low-dose light under a microscope. DOX/TPOR_4_ also has a stronger fluorescence intensity than TPOR alone under the same radiation conditions due to its ability to sensitize ROS. It is worth noting that among all test groups, DOX/TPOR_4_@CB[7]_4_ demonstrated enhanced photosensitivity and resulted in a significantly augmented cellular drug uptake by SH-SY5Y cells. Moreover, in line with the findings of the ^1^O_2_ assay, the inclusion of CB[7] led to a further enhancement in intracellular ROS generation. These findings suggest that treatment with DOX/TPOR_4_@CB[7]_4_ will have a more significant impact on tumor cell survival. Based on the host–guest interaction with CB[7], self-assembled compounds can effectively disperse and reduce TPOR accumulation under light excitation while producing ROS, which will have a significant tumor-killing effect [49,50]. Experimental results demonstrate that self-assembled DOX/TPOR_4_@CB[7]_4_ exhibits significantly enhanced optical properties, ROS yield, and improved drug stability. This suggests that CB[7] can enhance the fluorescence quantum yield of photosensitizers and contribute to their stability.

### 3.4. DOX/TPOR_4_@CB[7]_4_ Co-Locates in Mitochondria

The subcellular localization of DOX/TPOR_4_@CB[7]_4_ in SH-SY5Y cells was assessed by co-incubating DAPI as a nuclear stain and MitoTracker green as a mitochondrial marker for colocalization experiments. The emission fluorescence at 600–750 nm, corresponding to the inherent fluorescence properties of TPOR, was utilized to determine its cellular distribution. As shown in Figure 2C, TPOR, DOX/TPOR_4_, and DOX/TPOR_4_@CB[7]_4_ all exhibited predominantly cytoplasmic red fluorescence. The fluorescence intensity of DOX/TPOR_4_ was higher than that of TPOR due to the combination resulting in superimposed signals. Furthermore, the fluorescence intensity of DOX/TPOR_4_@CB[7]_4_ was significantly enhanced compared to that of DOX/TPOR_4_ and it displayed strong accumulation within SH-SY5Y cells. These findings suggest that DOX/TPOR_4_@CB[7]_4_, possessing suitable size and biocompatibility characteristics, efficiently entered the cells owing to TPOR’s specific affinity [51,52]. This indicates that under similar conditions, the addition of CB[7] improved the solubility of DOX/TPOR_4_ and facilitated drug uptake by SH-SY5Y cells. Moreover, from the Figure 2C, it can be observed that red fluorescence emitted by DOX/TPOR_4_@CB[7]_4_ showed good colocalization with MitoTracker green dye indicating a similar distribution pattern between drug molecules and mitochondria suggesting potential drug action on mitochondria. Under excitation by a 520 nm laser, DOX/TPOR_4_@CB[7]_4_ emits bright fluorescence in the range of 600–750 nm, which falls within the near-infrared region. This property facilitates tissue and cell penetration as well as easy observation within cells. The increased fluorescence emission in the combination group aids in visualizing tumor diagnosis and treatment.

### 3.5. Assessment of Phototoxicity, Dark Toxicity, and Potential Synergistic Effects

**Figure 3:**
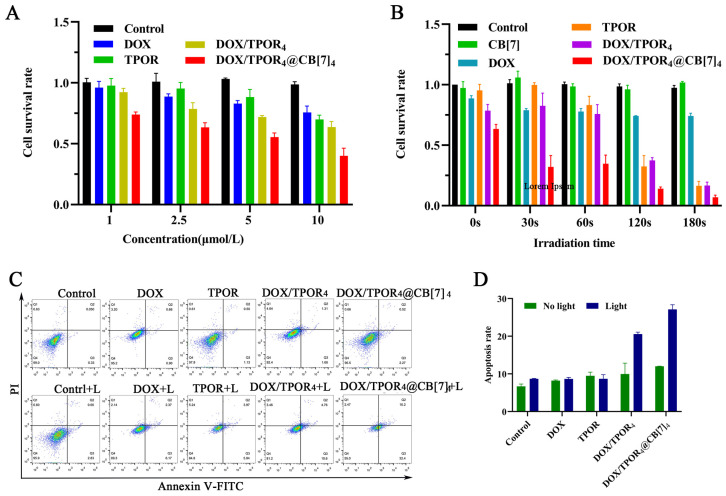
Killing effect of drugs on SH-SY5Y cells. (**A**) The cell survival rate of DOX, TPOR, DOX/TPOR_4_ and DOX/TPOR_4_@CB[7]_4_ groups without laser irradiation and (**B**) upon irradiation (525 nm, 95.5 mW/cm^2^) in SH-SY5Y cells measured by MTT (data are presented as mean ± SE (n = 3)). (**C**) Apoptosis rate of SH-SY5Y cells measured by flow cytometry. (**D**) The statistical analysis graph of apoptosis rate.

Previous experiments have demonstrated that CB[7] does not exert a significant inhibitory effect on the growth of SH-SY5Y cells within the concentration range of 1–10 μM [41]. As depicted in Figure S4, laser radiation employed in this study did not induce any notable inhibition on cell viability within the range of 0~250 mW/cm^2^, thereby excluding the influence of light radiation on cell survival. In the absence of light radiation, the cell survival rate was lower in the DOX/TPOR_4_ group compared to that in the DOX group at all concentrations, indicating that the chemotherapy drug DOX primarily contributed to cell death. The co-administration of DOX with TPOR enhanced DOX uptake, resulting in a more potent chemotherapy effect. Upon treatment with 1 μM DOX/TPOR_4_@CB[7]_4_, cellular viability rapidly decreased to approximately 74%, while significantly reducing the dosage of DOX as compared to the 10 μM DOX treatment group. Among all experimental groups, DOX/TPOR_4_@CB[7]_4_ exhibited cytotoxicity and further augmented its (DOX) efficacy as a chemotherapeutic agent. The inclusion of CB[7] substantially improved medication effectiveness. We propose that the co-administration of DOX and TPOR drugs can enhance the intracellular uptake of DOX in tumor cells due to the unique affinity exhibited by photosensitizer TPOR, thereby eliciting potentiated chemotherapeutic effects. As depicted in Figure 3B, the results demonstrated that laser irradiation did not significantly impact the efficacy of DOX, Under laser radiation, the experimental group containing TPOR exhibited a significant decrease in cell survival rate with an evident time-dependent effect on cell killing. After 30 s of laser irradiation, cell viability in the DOX/TPOR_4_@CB[7]_4_ group was approximately 33% (DOX: 84%, TPOR: 99%, DOX/TPOR_4_: 83%), indicating that DOX/TPOR_4_@CB[7]_4_ exhibited superior synergistic effects for photodynamic and chemotherapy in vitro. The combination drug exhibits significant potential in reducing the dosage of chemotherapy, thereby effectively mitigating systemic toxicity and treatment-related side effects, offering promising advantages for neuroblastoma treatment. The synergistic application of photodynamic therapy and chemotherapy can mutually enhance each other’s efficacy, surpassing the photodynamic effect observed in either TPOR group alone or DOX/TPOR_4_ group alone.

### 3.6. The Analysis of Flow Cytometry

To further assess the anti-tumor efficacy of the drug, we employed the ANNEXIN V-FITC/PI double labeling method to quantify the ratio of apoptosis to necrosis following PDT treatment [53]. As depicted in Figure 3C, in the absence of laser irradiation, no significant differences were observed in apoptosis compared to the control group. However, 4 h after 2 min of irradiation, both the DOX/TPOR_4_ group and DOX/TPOR_4_@CB[7]_4_ group exhibited significant rates of apoptosis at 20.93% and 28.0%, respectively. It is noteworthy that flow cytometry analysis of DOX/TPOR_4_@CB[7]_4_ yielded consistent results with the MTT assay, demonstrating pronounced cytotoxic effects on SH-SY5Y cells. The incorporation of CB[7] further augmented the synergistic effect between chemotherapy and phototherapy.

### 3.7. The In Vivo Assessment of the Antitumor Efficacy

**Figure 4:**
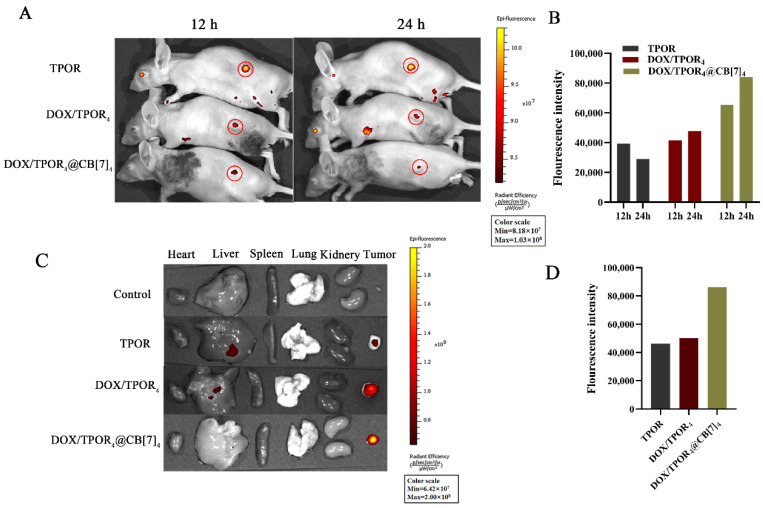
Images of drug biodistribution in mice. (**A**) Fluorescence images of SH-SY5Y tumor-bearing mice post intravenous injection of drugs for different times. (**B**) Quantification of fluorescence intensity of A. (**C**) Ex-vivo fluorescence images of isolated organs and tumor issue at 24 h post-injection of drugs. (**D**) Quantification of fluorescence intensity of C.

Encouraged by the results of in vitro cytotoxicity determinations, we further investigated the in vivo antitumor effect of synergistic chemotherapy combined with photodynamic therapy using SH-SY5Y cell xenograft models. Firstly, we performed in vivo tumor imaging and evaluated the accumulation behavior of DOX/TPOR_4_@CB[7]_4_ on an IVIS Spectrum system by utilizing the intrinsic fluorescence of TPOR. As depicted in Figure 4A,B, after 12 h of intravenous injection, fluorescence signals were observed in three groups of tumor tissues. Notably, a distinct fluorescence signal was captured after a 12 h intravenous injection of DOX/TPOR_4_@CB[7]_4_, which exhibited higher intensity after 12 h compared to both TPOR and DOX/TPOR_4_ groups, approximately twice as much. We observed that the fluorescence in the tumor tissue exhibited a gradual decrease subsequent to drug injection at 36 and 48 h (Figure S5). The peak fluorescence intensity persisted even at 24 h. Tumor tissue and major organs were harvested upon sacrificing mice at 24 h post-injection. Examination of the fluorescence images from these harvested materials revealed that DOX/TPOR_4_@CB[7]_4_ specifically emitted brighter signals within tumor tissue without any accumulation in healthy tissues (Figure 4C). This finding provides crucial evidence for visualizing the diagnosis and treatment of tumors. Loading CB[7] can effectively enhance tumor-targeting capability while reducing nonspecific capture by healthy organs and minimizing side effects.

**Figure 5:**
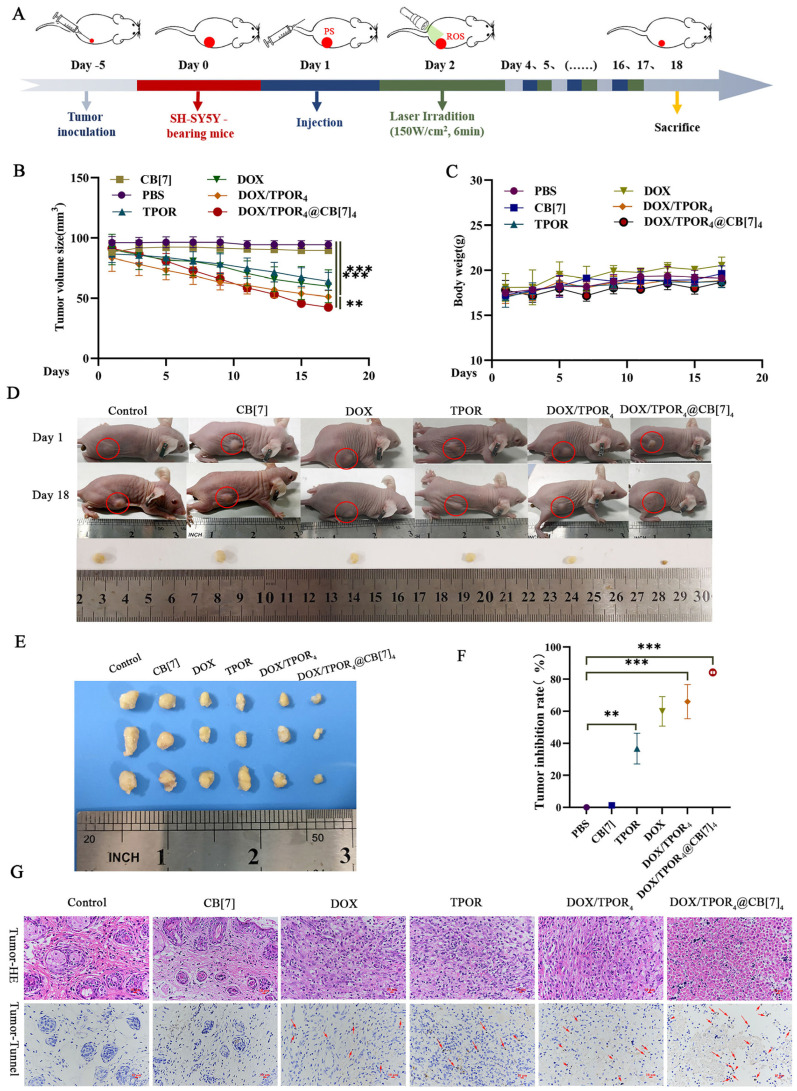
Inhibitory effect of drugs on tumor growth in tumor-bearing mice. (**A**) Flow chart of photodynamic therapy in mice; (**B**) tumor growth curves; (**C**) body weight curves; (**D**) photographs of tumor-bearing mice at day 0, 7 and 15 treated with different drugs (the red circle indicates the location of the tumor); (**E**) photographs of the tumor in vitro at the end of treatment; (**F**) tumor inhibition rate; (**G**) H&E staining and TUNEL staining of tumors after 18 days of treatments (The red arrow shows the positive result of TUNEL staining, ** *p* < 0.01, *** *p* < 0.001).

Furthermore, the efficacy of in vivo Chemo/PDT combination therapy was assessed by monitoring changes in tumor volumes and final tumor weight 18 days after different treatments (Figure 5A). After treatment, the DOX/TPOR_4_ group demonstrated superior anti-cancer efficiency compared to the DOX and TPOR groups. The DOX/TPOR_4_@CB[7]_4_ group exhibited a significantly greater reduction in tumor volume (Figure 5B), along with enhanced tumor inhibition (84.17%) compared to TPOR (36.69%) and DOX/TPOR_4_ (65.95%) groups (*p* < 0.001) (Figure 5E,F). Throughout the 18-day period, all groups maintained stable body weights without any observed skin damage on their surface skin during treatment on mice surface skin (Figure 5D).

**Figure 6:**
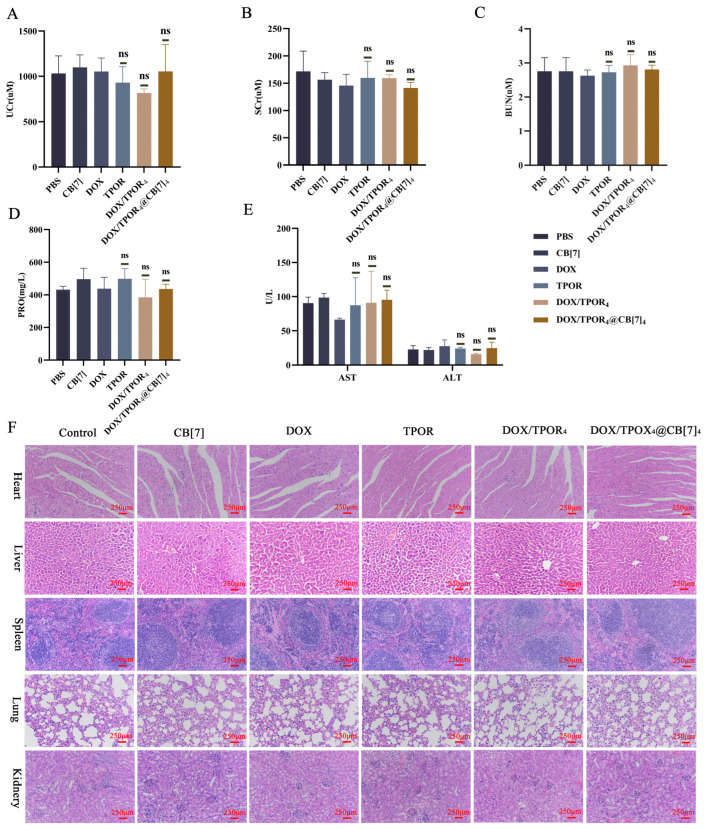
Biosafety analysis of different treatments. Content analysis of Serum renal function indicators Scr (**B**), BUN (**C**), urine Ucr (**A**), and PRO (**D**). (**F**) Content analysis of serum liver function indexes ALT, AST (**E**). Pathological analysis of major organs in different treatment groups after 18 days (**F**). Pathological analysis of major organs in different treatment groups after 18 days (F). (^ns^ *p* > 0.05).

Moreover, tumor slices were subjected to Hematoxylin and Eosin (H&E) staining assay. As depicted in Figure 5G, following an 18-day treatment period, the tumor tissue appeared normal in both the control and CB[7] groups. However, slight damage to the tumor tissue structure was observed in the DOX and TPOR groups. In contrast, evident cell damage accompanied by destruction of the tumor tissue structure was noted in the DOX/TPOR_4_ group. Notably, neovascularization and areas of tumor tissue necrosis significantly increased in the DOX/TPOR_4_@CB[7]_4_ group. Furthermore, TUNEL staining results demonstrated that apoptosis levels were highest in this particular group compared to others (Figure 6F). Subsequently, serum and urine samples from mice were analyzed for several crucial liver and kidney function indicators after treatment (Figure 6A–E), revealing no significant changes when compared with those of the control group for each indicator tested. Additionally, examination of tissue sections from major organs, including the heart, liver, spleen, lung, and kidney, indicated minimal damage within the treatment group as opposed to that observed within the control group further suggesting absence of any apparent biosafety concerns associated with this drug administration among mice.

The in vivo results have demonstrated that DOX/TPOR_4_@CB[7]_4_ exhibits superior tumor accumulation and prolonged fluorescence retention time, thereby enhancing the PDT and chemotherapy for an enhanced anti-tumor effect. This suggests that loading CB[7] improves the tumor-targeting ability and prolongs drug retention in the body, further enhancing the efficacy of TPOR and DOX combination therapy. Additionally, it can serve as a drug carrier to effectively reduce non-specific capture by healthy tissues while passively targeting tumors through enhanced permeability and retention effects, thus reducing toxic side effects. These findings suggest that DOX/TPOR_4_@CB[7]_4_ has no significant in vivo organ toxicity with potential as a potent therapeutic agent against cancer with minimal adverse effects.

## 4. Conclusions

To summarize our findings, we have successfully synthesized DOX/TPOR_4_@CB[7]_4_ to optimize the combination therapy involving chemotherapy and phototherapy. By exploiting TPOR’s specific affinity towards tumors, we observed an increased uptake of DOX by tumor cells, which consequently led to a reduction in the required dosage of DOX. Our assessment encompassing cell viability analysis, apoptosis rate determination, and an in vivo antitumor assay revealed heightened cytotoxicity of the drug on neuroblastoma cells, along with improved therapeutic outcomes through combined therapy. Consequently, this research offers valuable insights into CB[7] as a drug delivery carrier capable of addressing self-aggregation concerns while simultaneously augmenting the fluorescence intensity of photosensitizers via their self-assembly with CB[7], thus significantly enhancing tumor-targeting abilities. These discoveries are anticipated to establish a solid experimental basis for visual diagnosis and treatment strategies aimed at combating neuroblastoma.

## Data Availability

The original contributions presented in the study are included in the article/supplementary material; further inquiries can be directed to the corresponding author.

## Supplementary Materials

The following supporting information can be downloaded at: https://www.mdpi.com/article/10.3390/pharmaceutics16060822/s1, Figure S1. (A) TPOR; (B) DOX; Uv-vis absorption spectra (C) and fluorescence spectra (D) of DOX/TPOR_2_ with CB[7] in different proportions with the excitation of 520 nm; Uv-vis absorption spectra (E) and fluorescence spectra (F) of DOX/TPOR_4_ with CB[7]_4_ in different proportions with the excitation of 520 nm. (C_TPOR_ = 10.0 μmol/L, DMSO solution, 25 °C); Figure S2. Effects of different proportions of drugs on SH-SY5Y cells survival rate under photo-dynamic action (A: Control, B: CB[7], C: DOX, D: TPOR, E: DOX/TPOR_2_, F: DOX/TPOR_4_-CB[7]_2_, G: DOX/TPOR_4_-CB[7]_4_, H: DOX/TPOR_4_, I: DOX/TPOR_4_-CB[7]_2_, J: DOX/TPOR_4_-CB[7]_4_) (c: 2.5 μmol/L); Figure S3. The image of particle size distribution (A) and Zeta potential of TPOR, DOX/TPOR_4_ and DOX/TPOR_4_@CB[7]_4_ (B); Figure S4. Effect of 525 nm laser on the survival rate of SH-SY5Y cells; Figure S5. Images of drug biodistribution in mice. (A) Fluorescence images of SH-SY5Y tumor-bearing mice post intravenous injection of Drugs for different times; (B) Quantification of fluorescence intensity of A.
