# Supplementary material for: Self-Assembled Nanocomposite DOX/TPOR4@CB[7]4 for Enhanced Synergistic Photodynamic Therapy and Chemotherapy in Neuroblastoma
Source: Pharmaceutics. 2024 Jun 18;16(6):822. doi: 10.3390/pharmaceutics16060822 (PMC11207937; doi:10.3390/pharmaceutics16060822)
Supplement: Supplementary file 1 [file pharmaceutics-16-00822-s001.zip › pharmaceutics-3050525-supplementary.pdf]

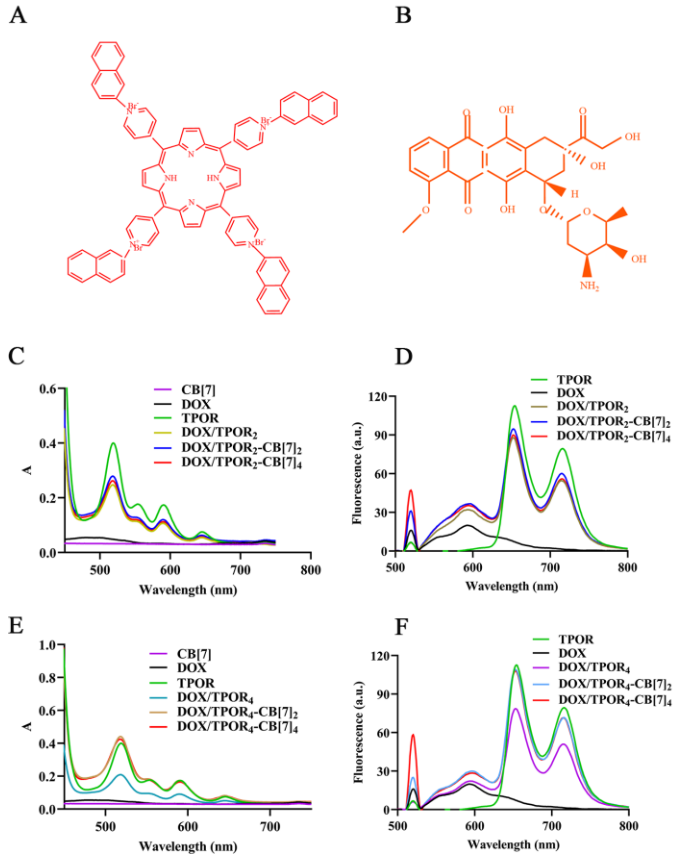

Figure S1. (A)TPOR;(B)DOX;Uv-vis absorption spectra (C) and fluorescence spectra (D) of DOX/TPOR$_2$ with CB[7] in different proportions with the excitation of 520 nm; Uv-vis absorption spectra (E) and fluorescence spectra (F) of DOX/TPOR$_4$ with CB[7]$_4$ in different proportions with the excitation of 520 nm.(C_{TPOR}=10.0 μmol/L, DMSO solution, 25°C)

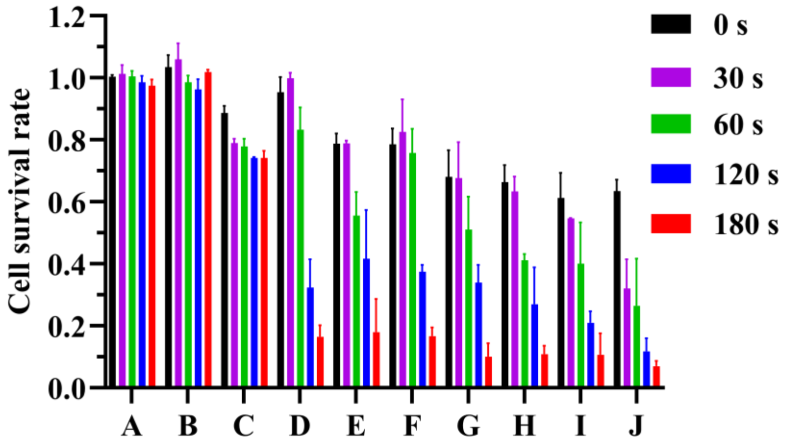

Figure S2. Effects of different proportions of drugs on SH-SY5Y cells survival rate under photodynamic action (A: Control, B: CB[7], C: DOX, D: TPOR, E: DOX/TPOR$_2$, F: DOX/TPOR$_4$-CB[7]$_2$, G: DOX/TPOR$_4$-CB[7]$_4$, H: DOX/TPOR$_4$, I: DOX/TPOR$_4$-CB[7]$_2$, J: DOX/TPOR$_4$-CB[7]$_4$) (c: 2.5 μmol/L).

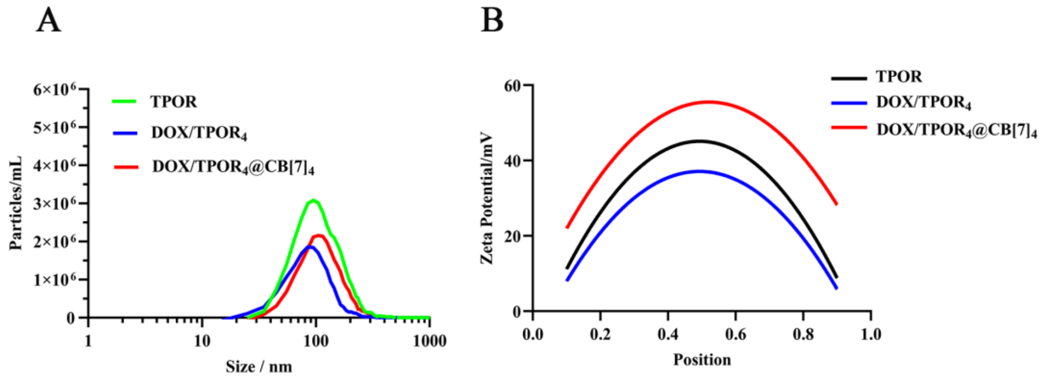

Figure S3. The image of particle size distribution(A) and Zeta potential of TPOR, DOX/TPOR4 and DOX/TPOR4@CB[7]4 (B).

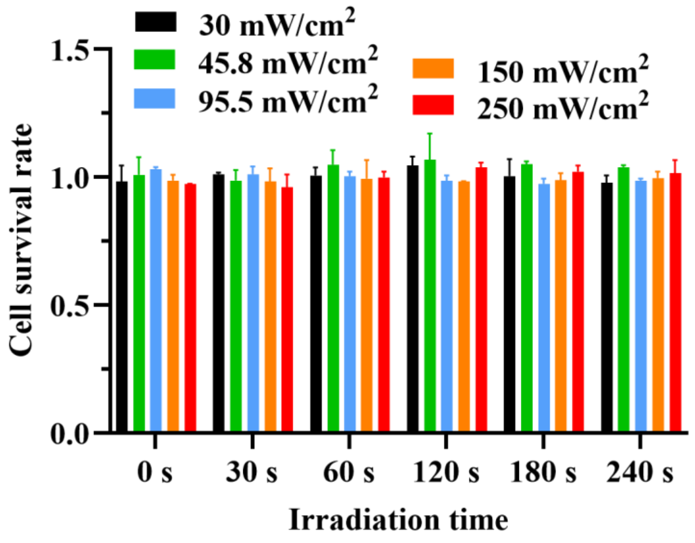

Figure S4. Effect of 525 nm laser on the survival rate of SH-SY5Y cells.

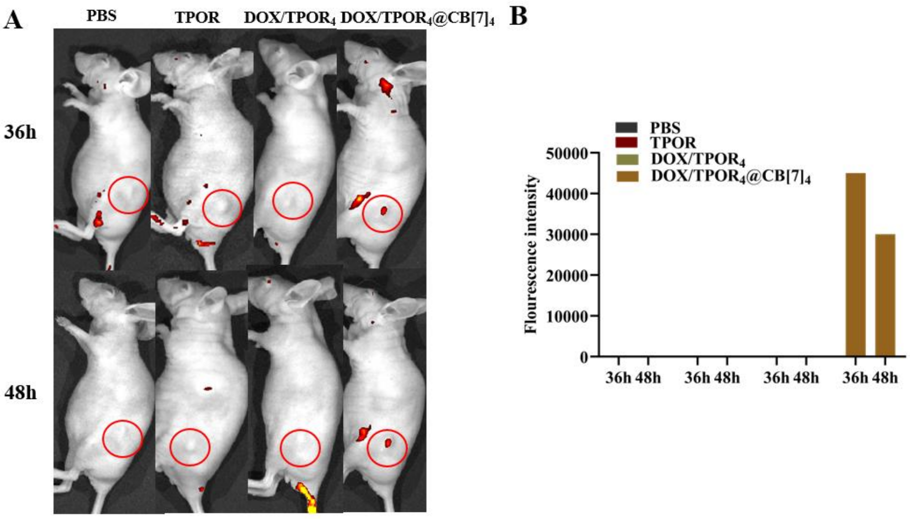

Figure S5. Images of drug biodistribution in mice. (A) Fluorescence images of SH-SY5Y tumor-bearing mice post intravenous injection of Drugs for different times; (B) Quantification of fluorescence intensity of A;
